# P024 High-risk antiphospholipid syndrome complicated by refractory immune thrombocytopenia

**DOI:** 10.1093/rap/rkag113.054

**Published:** 2026-11-03

**Authors:** Anzelika Karadzova-Stojanoska, Emilija Sekuloska, Mimoza Nikolovska-Kotevska, Sonja Pavlova, Sonja Vidinikj, Maja Bojadzioska, Sanja Petrova, Ana Vasilevska, Ron Vejseli, Martin Stojanoski

**Affiliations:** PHI University Clinic for Rheumatology, Skopje, North Macedonia, Republic of; PHI University Clinic for Rheumatology, Skopje, North Macedonia, Republic of; PHI University Clinic for Rheumatology, Skopje, North Macedonia, Republic of; PHI University Clinic for Rheumatology, Skopje, North Macedonia, Republic of; PHI University Clinic for Rheumatology, Skopje, North Macedonia, Republic of; PHI University Clinic for Rheumatology, Skopje, North Macedonia, Republic of; PHI University Clinic for Rheumatology, Skopje, North Macedonia, Republic of; PHI University Clinic for Rheumatology, Skopje, North Macedonia, Republic of; PHI University Clinic for Rheumatology, Skopje, North Macedonia, Republic of; PHI University Clinic for Hematology, Skopje, North Macedonia, Republic of

## Abstract

**Introduction:**

Mild thrombocytopenia is common in antiphospholipid syndrome (APS) due to overconsumption, while it may be severe in immune thrombocytopenia (ITP) because of antibody-mediated destruction. Antibody profiles may help in separating the two entities, but diagnosing the two together is still difficult as ITP can be hidden. Here we present the case of a 50-year-old woman presenting with symptoms of high-risk obstetric APS, only to be found out later to have refractory ITP as well, complicating treatment between coagulation and bleeding risk.

**Case description:**

A 50-year-old female patient with a history of five spontaneous abortions over a 5-year period, including a late fetal loss, was diagnosed with APS in 2011, with no criteria for other connective tissue diseases. She was lost to specialist follow-up and inconsistently treated with aspirin, LMWH, rivaroxaban, and acenocoumarol. On her labs, thrombocytopenia was consistent with values between 70 and 90. Hydroxychloroquine was initiated but due to worsening of her thrombocytopenia was discontinued. Over subsequent years she developed recurrent DVT, a cerebrovascular insult, and episodes of transient visual loss. She re-presented in 2025 with severe thrombocytopenia and extensive bruising, with immunological testing confirming positive lupus anticoagulant and anticardiolipin IgG, with no evidence of systemic connective tissue disease. Despite treatment with high-dose corticosteroids, three cycles of methylprednisolone pulses, hydroxychloroquine, and mycophenolate mofetil she demonstrated persistent cyclic thrombocytopenia with poor therapeutic response. Anticoagulation posed a major challenge with LMWH being withheld when platelets fell below 20, and acenocoumarol therapy complicated by marked INR instability (0.9-4.4). It was concluded that she had primary APS with coexisting steroid-refractory ITP, requiring referral to hematology for advanced therapies. The patient continues her treatment with mofetil mycophenolate, hydroxychloroquine, low dose corticosteroids and acenocoumarol depending on her INR and platelet count. Platelets continue to vary between 30 to a maximum of 130.

**Discussion:**

This case illustrates the difficulty of managing high-risk APS when complicated by true ITP. Thrombocytopenia was initially attributed to APS, but later development raised suspicion for coexisting ITP, this distinction is clinically crucial because APS-related thrombocytopenia is usually mild, whereas ITP requires targeted immunosuppression. aPL antibodies can appear in ITP without true APS. In APS thrombocytopenia is typically consumptive while in ITP it is destructive and often more severe, coexistence increases both bleeding and thrombotic risk. Management required constant balancing between thrombosis and bleeding, with anticoagulation being withheld when platelets fell below 20, exposing the patient to significant thrombotic risk given her high-risk APS profile. Attempt at immunosuppression failed to stabilise platelet counts, necessitating consideration of advanced therapies such as IVIG or rituximab. This case underscores the need for early recognition of overlapping autoimmune hematologic disorders, aggressive management of high-risk APS, and strict adherence to long-term follow-up. Failure to maintain consistent anticoagulation and immunosuppression, as with this patient, can lead to preventable morbidity.

**Key learning points:**

• Balancing treatment of thrombosis versus bleeding risk requires utmost vigilance and often demanding multidisciplinary decision-making. Severe thrombocytopenia should not be attributed to APS itself, as ITP can coexist, which requires separate treatment methods. Inadequate and inconsistent anticoagulation such as in this case leads to major thrombotic events such as DVT and stroke. With platelets remaining critically low, immunosuppressive therapy is needed such as mycophenolate, IVIG or rituximab, especially in steroid-refractory thrombocytopenia. The need for structured long-term management and patient education is highlighted as inconsistent therapy and irregular follow-up can significantly worsen APS outcomes.

